# Supplementary material for: Development, feasibility and potential effectiveness of community-based continuous mass dog vaccination delivery strategies: Lessons for optimization and replication
Source: PLoS Negl Trop Dis. 2022 Sep 6;16(9):e0010318. doi: 10.1371/journal.pntd.0010318 (PMC9481168; doi:10.1371/journal.pntd.0010318)
Supplement: S1 Table — (DOCX) [file pntd.0010318.s001.docx]

S1 Table: Delivery of 45 components CBC-MDV compared to what was planned.

| **CBC-MDV COMPONENTS** | **LEVEL OF FIDELITY TO IMPLEMENTATION PROTOCOL** | | | |
| --- | --- | --- | --- | --- |
|  | Delivered as planned (44%) | Not delivered as planned (31%) | Modified/ partly delivered (20%) | Delivered in excess of what was planned (5%) |
| 1. **Local delivery of CBC-MDV to be led by district level veterinary authorities** | | | | |
| District veterinary office to receive and manage stocks of vaccination materials | 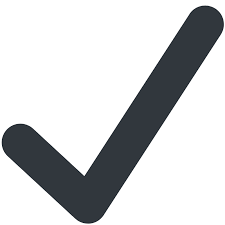 |  |  |  |
| District veterinary office will coordinate transport of materials to wards and ensure unused vaccines after six months are returned and properly labeled for storage |  | 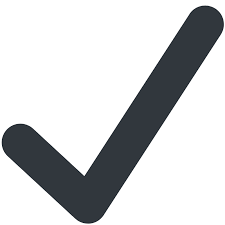 |  |  |
| District veterinary office to be responsible for monitoring implementation of CBC-MDV components |  | 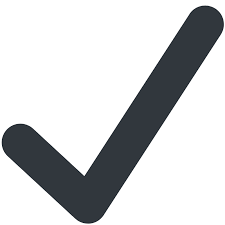 |  |  |
| District veterinary office to introduce village-based One Health Champions (OHCs) to village leadership with letter before campaign starts | 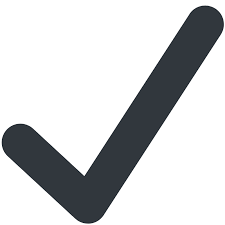 |  |  |  |
| 1. **Involvement of village level leadership in roll out of CBC-MDV** | | | | |
| The OHCs to introduce themselves to village leaders using a letter from District veterinary office |  |  | 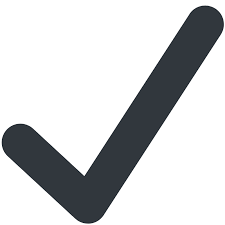 |  |
| OHC to sensitize leadership of villages, schools, churches, mosques, neighborhood groups, NGOs/firms (if any) on: burden of rabies, benefits of mass dog vaccination, the campaign and their expected roles; 7-10 days before campaigns begin |  |  | 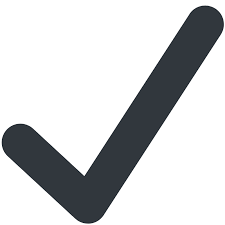 |  |
| OHCs and RCs to draw timetable and communicate plans to village chairman/executive officers, get permission to advertise campaigns |  |  | 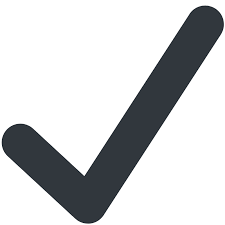 |  |
| OHCs and RCs to ask village officers to encourage/mobilize villagers to bring their dogs for vaccination |  |  | 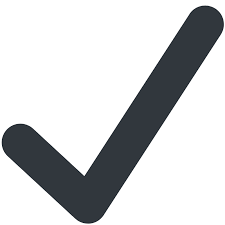 |  |
| OHC to request estimate of dogs in ten-cell compartments of village from ‘mabalozi’ (leaders of) and send total for the village to RC before campaign |  | 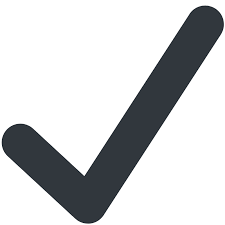 |  |  |
| 1. **Use of trained village-based One Health Champions to support ward-level rabies coordinators to carry out vaccination activities** | | | | |
| OHCs will estimate dog population of their respective villages | 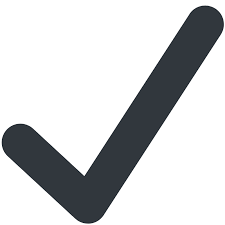 |  |  |  |
| OHC to sensitize villagers about forthcoming vaccination clinics at village meetings |  |  | 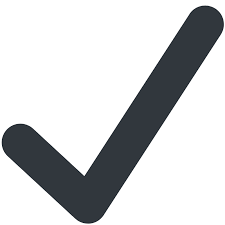 |  |
| OHCs to visit houses after each round and compile a list of dogs that missed vaccination and plan with RC to vaccinate them, also document pregnant dogs |  | 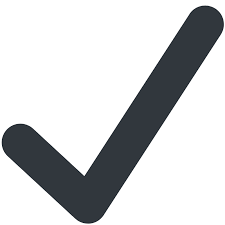 |  |  |
| OHCs to give their telephone number out to villagers (during sensitization meetings, advertising and clinic days) to call them anytime if they have concerns about rabies or if they have new dogs or puppies that need to be vaccinated in between campaigns |  | 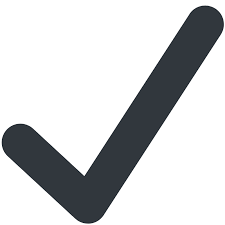 |  |  |
| OHCs to advertise vaccination clinic using posters, loud speaker or word of mouth | 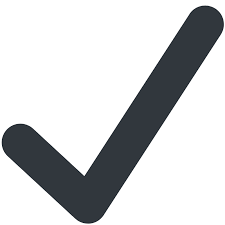 |  |  |  |
| On day of vaccination OHC will assist RC by entering dog data into register, issue vaccination certificates, restraining of dogs and putting on of collar where necessary |  |  |  | 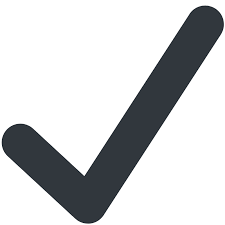 |
| 1. **Widespread communication at village level about CBC-MDV and advertising of campaigns using multiple forms of communication and venues** | | | | |
| Advertising of campaigns to be done a day before |  |  |  | 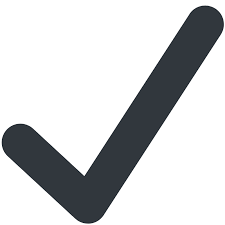 |
| Advertising of vaccination clinics to made at popular places like schools, market squares, churches and mosques, and village meetings | 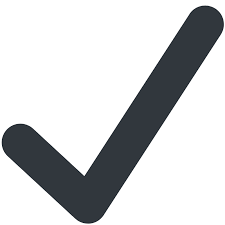 |  |  |  |
| OHCs and RCs to inform targeted households for house-to-house campaigns through ‘mabalozi’ (leaders of cluster of ten houses) |  |  | 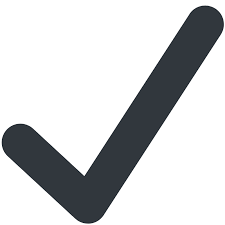 |  |
| 1. **Use of locally designed cooling clay pots to store rabies vaccine in wards** | | | | |
| Rabies Coordinators (RCs) to send request for materials, accompanied with dog population estimate for ward, via sms, then call district veterinarian to schedule a day for pick up | 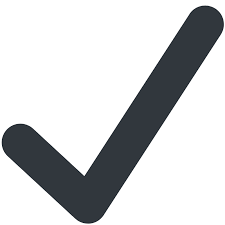 |  |  |  |
| RCs to liaise with DLFO to coordinate transport of new batches of vaccines from district office to ward | 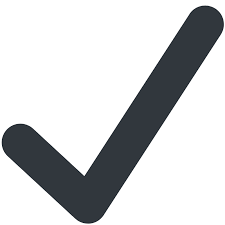 |  |  |  |
| Equipment and vaccines will be requested based on dog population of ward | 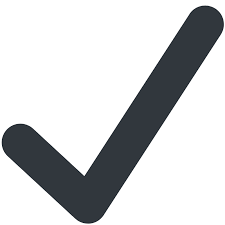 |  |  |  |
| RCs to store batches of vaccines in cooling pots on six monthly bases to ensure vaccines that have stayed outside of the cold chain for more than six months are not used | 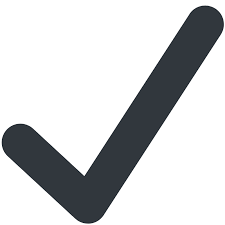 |  |  |  |
| RCs to return unused vaccines at the end of the sixth month period to DLFDO’s office to be labelled ‘X’ with a black marker pen and stored |  | 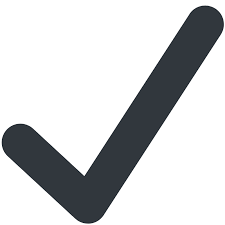 |  |  |
| RCs to return used needles and microchip units to DLFDO’s office or health center for proper disposal |  |  | 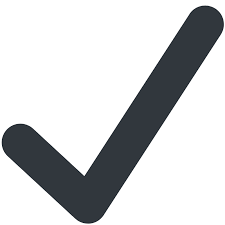 |  |
| RCs to place cooling pot in the appropriate place at home, add water regularly | 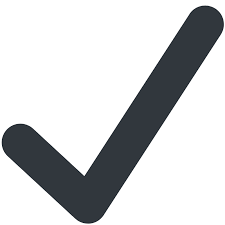 |  |  |  |
| RCs to monitor and record temperature of pot daily | 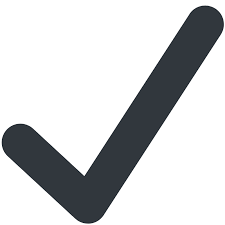 |  |  |  |
| 1. **A continuous approach to MDV activities; quarterly basis and available on demand by dog owners all year round** | | | | |
| Vaccinators were to organize quarterly vaccination campaigns: Central point campaign strategy by all arms during month 1 vaccination |  |  | 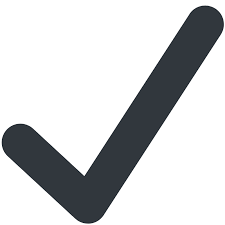 |  |
| During subsequent rounds, vaccinators can adopt house-to-House approach to reach more dogs | 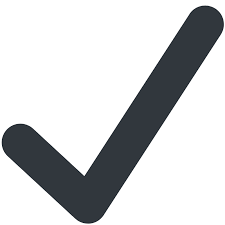 |  |  |  |
| Throughout the year, teams will provide vaccination services to owners on-demand: vaccinators can either visit owner or ask owner to bring dog upon call from owner | 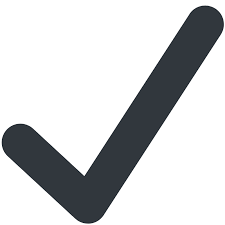 |  |  |  |
| Subsequent to each round, OHCs will go round their villages to document dogs the missed the previous round to be targeted for vaccination |  | 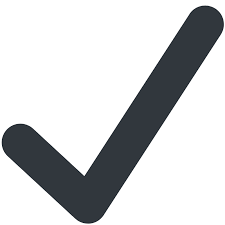 |  |  |
| The vaccinators were to ensure villagers have continuous access to vaccination by giving out their telephone contacts |  | 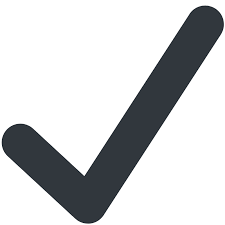 |  |  |
| 1. **Delivery of free dog vaccination clinics using suitable approaches** | | | | |
| Vaccination clinics were to start from 08 HOURS and end 14 HOURS |  |  | 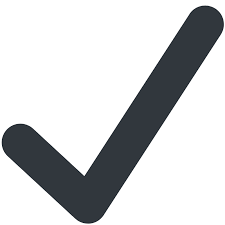 |  |
| Registration center set approximately 20 meters apart from the inoculation and microchipping center |  | 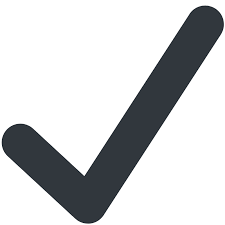 |  |  |
| Team to use muzzles on potentially aggressive dogs |  | 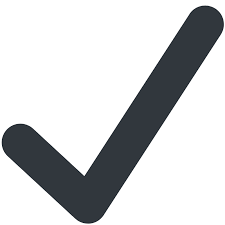 |  |  |
| Dog owners to stand in queues to ensure first-come first-served and to reduced fighting among dogs | 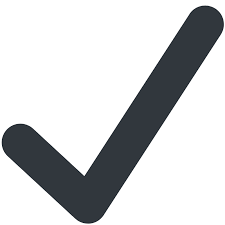 |  |  |  |
| Dog owners to leave immediately with their dogs after vaccination to avoid crowding of dogs | 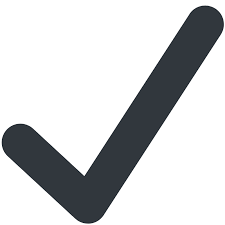 |  |  |  |
| All animals vaccinated will have their biodata entered into a local register (to be kept at ward level) and an online database, certified, microchipped and collared | 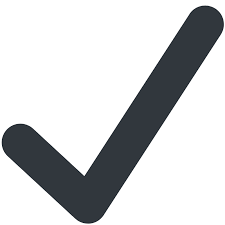 |  |  |  |
| Vaccination of dogs will be delivered free of charge | 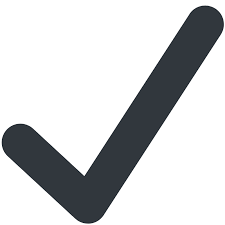 |  |  |  |
| Vaccination team to deliver subsequent campaigns using approaches they deemed appropriate | 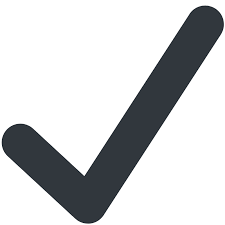 |  |  |  |
| 1. **Monitoring and feedback on vaccination coverage among research team, district veterinary authorities, vaccinators and communities** | | | | |
| District veterinary office to supervise and monitor implementation of campaigns |  | 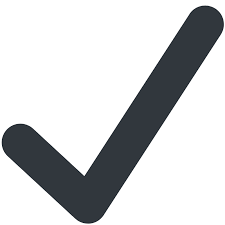 |  |  |
| OHCs to prepare weekly reports on dogs needing vaccination and other issues to be discussed with RC in weekly report |  | 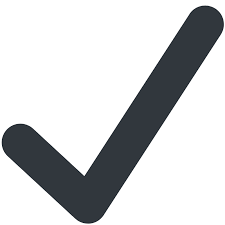 |  |  |
| Research team to give feedback to communities after first round of data collection |  | 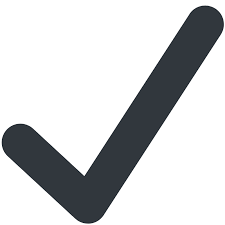 |  |  |
| A 3-member committee selected by the village executive to conduct community self-monitoring to see if RCs, OHCs and villagers are working together to ensure all dogs are vaccinated |  | 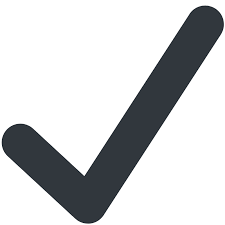 |  |  |
| OHCs to conduct transect after the day’s clinic to access vaccination coverage | 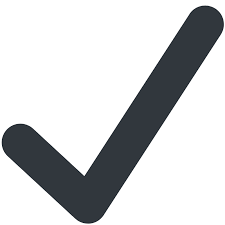 |  |  |  |
| RCs to provide summary reports on number of animals vaccinated, temperature of cooling pot and rabies events to DLFDO’s office/research team via WhatsApp | 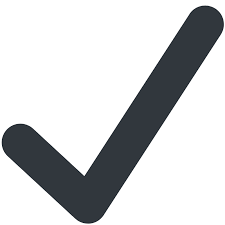 |  |  |  |

*OHCs: One Health Champions, *MDV: Mass Dog Vaccination, *RCs: Rabies Coordinators
