# Supplementary material for: Development, feasibility and potential effectiveness of community-based continuous mass dog vaccination delivery strategies: Lessons for optimization and replication
Source: PLoS Negl Trop Dis. 2022 Sep 6;16(9):e0010318. doi: 10.1371/journal.pntd.0010318 (PMC9481168; doi:10.1371/journal.pntd.0010318)
Supplement: S2 Table — (DOCX) [file pntd.0010318.s002.docx]

Table B in S1 Text: Comparison of fidelity and reasons for variations in delivery of components of CBC-MDV by strategy arms.

| **CBC-MDV COMPONENTS** | **Strategy 1** | **Strategy 2** | **Strategy 3** | Total No. of teams (*N*=9)/ One Health Champions (*N*=35) who delivered component |
| --- | --- | --- | --- | --- |
|  | No. of teams (*N*=3)/ One Health Champions (*N*=12) who delivered component | No. of teams (*N*=3)/ One Health Champions (*N*=13) who delivered component | No. of teams (*N*=3)/ One Health Champions (*N*=10) who delivered component |  |
| **Involvement of village level leadership in roll out of CBC-MDV** | | | | |
| The OHCs who introduced themselves to village leaders using a letter from District veterinary office | 8 | 13 | 10 | 31 |
| OHC who sensitized leadership of villages, schools, churches, mosques, neighborhood groups, NGOs/firms (if any) on: burden of rabies, benefits of mass dog vaccination, the campaign and their expected roles; 7-10 days before campaigns begin | 0 | 0 | 0 | 0 |
| RCs who discussed their timetable with village chairman/ executive officers, get permission to advertise campaigns | 0 | 2 | 2 | 4 |
| OHCs who asked village officers to encourage/ mobilize villagers to bring their dogs for vaccination | 2 | 8 | 9 | 19 |
| OHCs who requested estimate of dogs in ten-cell compartments of village from ‘mabalozi’ (leaders of cluster of ten houses) and send total for the village to RC before campaign | 3 | 0 | 0 | 3 |
| **Use of trained village-based One Health Champions to support ward-level rabies coordinators** | | | | |
| OHCs who estimated dog population of their respective villages | 12 | 13 | 10 | 35 |
| OHCs who sensitized villagers about forthcoming vaccination clinics at village meetings | 2 | 8 | 9 | 19 |
| OHCs who advertised vaccination clinic using posters, loud speaker or word of mouth | 12 | 13 | 10 | 35 |
| OHCs who assisted RCs on day of vaccination entering dog data into register, issue vaccination certificates, restraining of dogs and putting on of collar where necessary | 12 | 13 | 10 | 35 |
| **Advertising of campaigns** | | | | |
| OHCs who advertised vaccination campaigns a day before | 12 | 13 | 10 | 35 |
| OHCs who advertised vaccination clinics at popular places like schools, market squares, churches and mosques | 12 | 13 | 10 | 35 |
| Teams who informed targeted households for house-to-house campaigns through ‘mabalozi’ (leaders of cluster of ten houses) | - | - | - | - |
| **Use of locally designed cooling clay pots to store rabies vaccine in wards** | | | | |
| RCs who sent request for materials, accompanied with dog population estimate for ward, via sms, then call district veterinarian to schedule a day for pick up | 3 | 3 | 3 | 9 |
| RCs who liaised with DLFO to coordinate transport of new batches of vaccines from district office to ward | 3 | 3 | 3 | 9 |
| RCs who requested equipment and vaccines based on 80% of dog population of ward | 3 | 3 | 3 | 9 |
| RCs who stored batches of vaccines in cooling pots on six monthly bases to ensure vaccines that have stayed outside of the cold chain for more than six months are not used | 3 | 3 | 3 | 9 |
| RCs who returned unused vaccines at the end of the sixth month period to DLFDO’s office to be labelled ‘X’ with a black marker pen and stored | 1 | 1 | 0 | 2 |
| RCs who returned used needles and microchip units to DLFDO’s office or health center for proper disposal | 2 | 2 | 1 | 5 |
| RCs who placed cooling pot in the appropriate place at home, added water regularly | 3 | 3 | 3 | 9 |
| RCs who monitored and recorded temperature of pot daily | 3 | 3 | 3 | 9 |
| **Providing continuous access to dog vaccination** | | | | |
| Teams who organized quarterly vaccination campaigns: Central point campaign strategy by all arms during month 1 vaccination | 9 out of 12 | 11 out of 12 | 9 out of 12 | 29 out of 35 |
| Number of days vaccinators adopted House-to-House approach to reach more dogs during subsequent rounds | 3 days of HTH | 10 days of HTH | 15 days of HTH | - |
| Number of days throughout the year that teams provided vaccination services to owners on-demand: either visited owner or asked owner to bring dog upon call from owner | 7 days of on-demand | 8 days of on-demand | 5 days of on-demand | - |
| OHCs who visited houses after each round and compiled lists of dogs that missed vaccination and planned with RC to vaccinate them, also documented pregnant dogs | Not done | Not done | Not done | - |
| OHCs who gave their telephone number out to villagers (during sensitization meetings, advertising and clinic days) to call them anytime if they have concerns about rabies or if they have new dogs or puppies that need to be vaccinated in between campaigns | Not done | Not done | Not done | - |
| **Delivery of free dog vaccination clinics using suitable approaches** | | | | |
| Vaccination clinics were to start from 08 HOURS and end 14 HOURS | Varied start and closing time | Varied start and closing time | Varied start and closing time | - |
| Teams who set registration center approximately 20 meters apart from the inoculation and microchipping center | Not done | Not done | Not done | - |
| Teams who muzzled on potentially aggressive dogs | Not done | Not done | Not done | - |
| Dog owners to stand in queues to ensure first-come first-served and to reduce fighting among dogs | Delivered as planned | Delivered as planned | Delivered as planned | - |
| Dog owners to leave immediately with their dogs after vaccination to avoid crowding of dogs | Delivered as planned | Delivered as planned | Delivered as planned | - |
| All animals vaccinated will have their biodata entered into a local register (to be kept at ward level) and an online database, certified, microchipped and collared | Delivered as planned | Delivered as planned | Delivered as planned | - |
| Vaccination of dogs will be delivered free of charge | Delivered as planned | Delivered as planned | Delivered as planned | - |
| Vaccination team to deliver subsequent campaigns using approaches they deemed appropriate | Delivered as planned | Delivered as planned | Delivered as planned | - |
| **Monitoring and feedback on vaccination coverage among stakeholders** | | | | |
| RCs who reported district veterinary office supervised and monitored implementation of campaigns | Not done | Not done | Not done | - |
| OHCs who prepared weekly reports on dogs needing vaccination and other issues to be discussed with RC in weekly report | Not done | Not done | Not done | - |
| Research team to give feedback to communities after first round of data collection | Not done | Not done | Not done | - |
| A 3-member committee selected by the village executive to conduct community self-monitoring to see if RCs, OHCs and villagers are working together to ensure all dogs are vaccinated | Not done | Not done | Not done | - |
| OHCs to conduct transect after the day’s clinic to access vaccination coverage | 12 | 13 | 10 | 35 |
| RCs to provide summary reports on number of animals vaccinated, temperature of cooling pot and rabies events to DLFDO’s office/research team via WhatsApp | 3 | 3 | 3 | 9 |
