# Supplementary material for: Development, feasibility and potential effectiveness of community-based continuous mass dog vaccination delivery strategies: Lessons for optimization and replication
Source: PLoS Negl Trop Dis. 2022 Sep 6;16(9):e0010318. doi: 10.1371/journal.pntd.0010318 (PMC9481168; doi:10.1371/journal.pntd.0010318)
Supplement: S3 Table — (DOCX) [file pntd.0010318.s003.docx]

Table C in S1 Text: Utility of approaches and number of rounds of vaccination clinics organized by strategy arms.

| Strategy Arms | How vaccination clinics were organized by strategy arms during rounds of campaigns | | | |
| --- | --- | --- | --- | --- |
|  | **Round-1: Month-1** | **Round-2: Month-3** | **Round-3: Month-6** | **Round-4: Month-9** |
| Strategy One  Buswahili | A-day village level temporal static point clinics for 4 villages | A-day village level temporal static point clinics for 3 villages: 2 days for 1 village with many dogs | A-day village level temporal static point clinics for 3 out of 4 villages + 3 on-demand | No campaign activity |
| Strategy One  Tai Ward | A-day village level temporal static point clinics for 4 villages | 7, a-day temporal static point clinics for selected subvillages | No campaign activity | 1day temporal static point clinics for 2 subvillages + 2 on-demand |
| Strategy One  Gorong’a Ward | A-day village level temporal static point clinics for 3 villages: 2 days for 1 village with many dogs  Team then followed with 9, a-day temporal static point clinics for single or combined subvillages after 3 weeks | 7, a-day temporal static point clinics for selected subvillages | 3 days house-to-house for scattered houses + temporal static point clinics for clustered houses + 2 on-demand | No campaign activity |
| Strategy Two  Sirorisimba Ward | 14, a-day Subvillage level temporal static point clinics | 7, a-day temporal static point clinics for single or combined subvillages | 6, a-day temporal static point clinics for single or combined subvillages | 2, a-day temporal static point clinics for single or combined subvillages + 3 on-demand |
| Strategy Two  Mkoma Ward | 20, a-day Subvillage level temporal static point clinics | 6 village level temporal static point clinics | 2-day house-to-house campaigns + 3 on-demand | No campaign activity |
| Strategy Two  Nyanungu Ward | 27, a-day Subvillage level temporal static point clinics | 6 days mixed village, subvillage level temporal static point, house-to-house clinics + 2 on-demand | 4-day house-to-house campaigns | A-day house-to-house campaign |
| Strategy Three  Nyamemange Ward | 8, 2 combined subvillages level temporal static point clinics per day | 6, 2 combined subvillages level temporal static point clinics per day | No campaign activity | No campaign activity |
| Strategy Three  Bukura Ward | 35, a-day subvillage level temporal static point clinics | 5 village level temporal static point clinics | 5-day house-to-house campaigns + 4 on-demand | No campaign activity |
| Strategy Three  Itiryo Ward | 15, a-day subvillage level temporal static point clinics | 12 days mixed subvillage level temporal static point & house-to-house clinics | 7 days mixed subvillage level temporal static point & house-to-house clinics | A-day house-to-house campaign + 1 on-demand |

*A day refers to one vaccination clinic period, planned for 08:00 -14:00 Hours
