# Supplementary material for: Development, feasibility and potential effectiveness of community-based continuous mass dog vaccination delivery strategies: Lessons for optimization and replication
Source: PLoS Negl Trop Dis. 2022 Sep 6;16(9):e0010318. doi: 10.1371/journal.pntd.0010318 (PMC9481168; doi:10.1371/journal.pntd.0010318)
Supplement: Attachment — Submitted filename: renamed_56072.docx [file pntd.0010318.s004.docx]

Dear Editors,

**Response to yours and reviewers’ comments**

Development, feasibility and potential effectiveness of community-based continuous mass dog vaccination delivery strategies: lessons for optimization and replication (PNTD-D-22-00320).

Thank you very much for the opportunity to respond to reviewers’ comments in a revised submission of our manuscript. We thank the reviewers for constructive comments which we have now addressed, and we consider have resulted in an improved version of the manuscript. Page numbers refer to those in the revised manuscript with tracked changes.

Reviewer #1

| **Comment** | **Response** |
| --- | --- |
| General: Please check the spelling consistency between US and UK English throughout. E.g., line 350, the word “programme” (UK English) is used, while line 383, “center” (US English) is used. | Thank you for prompting us on the inconsistent spelling of those words. Consistency between US and UK English was checked for words like: “programme”, “center”, “analyzed”, “summarized” and one form of the words are used throughout the manuscript |
| Table 3. Point v, third column: The words “in locally made” are repeated. Please correct. | Thank you for pointing out the error, one of “locally made” is removed |
| Line 361: Please change “seen” to “saw”. The grammar of the sentence can be improved by saying, “or did not see the project…”. | Thank you for the recommendation, we changed “seen” to “did not see”, line 376 |
| Line 384: “Not all of OHCs had cooperation…” Please either delete the “of” or include “of the”. | Thank you for pointing out the error, “of” is now deleted, line 398 |
| Line 394: Mega phones – please correct to megaphones (one word). | Thank you for pointing out the error, “mega phones” is changed to “megaphones”, line 408 |
| Line 413: Please replace “is” to “was”. | Thank you for pointing out the error, “is” is changed to “was”, line 427 |
| Line 495 – 497: Three things are listed here. What do you mean by "Rabies events"? Are these potential rabies cases or are they vaccination clinics? Please clarify. | Thank you for pointing out the error, "Rabies events" is changed to "Rabies cases", line 510 |
| Line 506 and 507: This is a section heading, whilst line 507 is a sub-heading – the sizing of this is the same and therefore makes it challenging to understand. Please ensure that this is clear for the final editorial process to distinguish the two clearly. | Thank you for pointing out the ambiguity, it is now distinguished by italicizing the subheadings, lines 520-522 |
| Line 543: “The Strategy teams partly followed CBC-MDV manual…” Please include “the” or make “Manual” plural. | Thank you for pointing out the ambiguity, “the” is now included, line 565 |
| Line 609: “and increase community support and contributions” Please include the word “can” or a similar grammatical revision of the sentence. | Thank you for pointing out the ambiguity, “potentially could have led to increased” is added to clarify the statement, line 638 |
| Line 619: It is mentioned that “information recording” was carried out with high-fidelity, yet in lines 500-502, it is highlighted that reporting load was too high and not done according to protocol. I assume that “information recording” relates to the “reporting on dogs vaccinated” – if this is the case, then please specify. | Thank you for pointing out the ambiguity, the statement was specified by making read “OHCs also did not provide weekly tallies of dogs needing vaccination, and they considered the weekly submission of tallies too frequent to allow for completion”, lines 514-516 |
| Line 673 – 674: “account for 3 out of the 7 missed rounds by all strategies and recording a lower coverage at month 11.”  - The sentence is a little ambiguous as it relates to the comparison with other strategies but can also be interpreted as a lower coverage when compared with month 1 of strategy III (as the authors assumedly intended). If a comparison of the vaccination coverages in month 11 is done, Strategy III is only lower than Strategy II. It is higher than Strategy I and the Pulse strategy. | Thank you for pointing out the ambiguity, the statement was reworded to clarify the point, and it now reads “However, the discretion also may have caused strategy 3 teams to relax after the first round of clinics, as they account for 3 out of the 7 missed rounds by all strategies and could be why they recorded a lower coverage at month 11”, lines 724-727 |
| Line 711: Please correct “Delivery” to “Deliver”. | Thank you for pointing out the error, “Delivery” was removed, line 780 |
| Line 723: The word “dose” has no context. Are the authors referring to the delivery of more vaccine doses? Please consider and clarify. | The statement was reworded to clarify the point, and it now reads “Overall, we conclude that improved supervision and monitoring, as well as community participation in designing, planning and implementing of the dog vaccination campaigns could result in higher fidelity and reach of the CBC-MDV strategies in a more sustainable manner”. line 789-793 |
| Supplementary material: Table S2, sub-heading “Providing continuous access to dog vaccination”, first row: The total listed here is 36. Please correct. | Thank you for pointing out the error, it is corrected from 36 to 35 |
| Table S2, sub-heading “Delivery of free dog vaccination clinics using suitable approaches”, first row: Spelling mistake “Start”. | Thank you for pointing out the error, the word “tart” is replaced with “start” |
| Table S3: Please clarify as to what “A-day” is? | Thank you for pointing out the ambiguity, the clarification, “A Day refers to one vaccination clinic period, planned for 08:00 -14:00 hours”, was added |
| Table S3: 9 wards are mentioned here of the 12 wards from each district (i.e., 36 wards across 3 districts?). I assume the other 3 wards for this particular district were the Pulse campaign? When was that campaign undertaken and how? Where are the data for the other 2 Districts? (refer to lines 164 - 167 in the main text and the earlier comment). | Thank you for pointing out the ambiguity. The following clarification was added “The pilot phase included 12 wards, four from each district (three delivered the CBC-MDV strategies and one delivered the pulse)”, Lines 172-173.  The CBC-MDV and pulse campaigns were carried out over the same time period” (Lines 325-326) to clarify the statement. |
| 1) It would be helpful to have a better understanding of the scale of the various administrative levels. For instance, how big is a ward (on average), and then within that, how big is a village, sub-village. What is the distance between sub-villages? Later in the results for instance, the authors mention that the OHC did not work with the Mabalozi who are responsible for 10 households, and rather worked with sub-village leaders. Approximately how many households are in a sub-village – would this be two mabalozi, or would it be 20? This could perhaps have an influence as to why the sub-village leaders were rather approached (in addition to the reasons provided). This would also help to clarify the challenges and fatigue faced by RCs and OHCs when working at the sub-village level, as the distance between sub-villages for example, have not been shared with the reader. | Thank you for your recommendation.  We have included a map to show locations of the districts and wards, and surface areas of the villages to show how much distance OHC had to cover during their work (Fig 2).  Approximately, subvillages comprise of 70-100 households, lines 176-177  Current information (for example shapefiles, number of households) on sub-villages will become available if collected in the ongoing census, and after compilation by Tanzania National Bureau of Statistics. |
| 2)      How many animals were vaccinated in each sub-village/village/ward using the different methods. While vaccination coverage is a good indicator in terms of controlling the disease, it is not suitably descriptive when comparing the efficacy of different methods, as the dog population in one village/ward may be significantly greater than in another, thus skewing the interpretation of the results. | Thank you for your observation and recommendation. The complete report on vaccination outcome indicators was reported in another paper, Lugelo et al., 2022 and duly referenced in this paper. This process evaluation paper focuses on the process and hence only provides summary of outcome results to give context to conclusions made on the processes to give readers appreciation of the possible implications of the process findings on outcome indicators |
| 3)      One of the main limitations listed for a “Pulse” vaccination campaign is the logistical costs and need for vehicles and fuel to deliver the annual vaccination. However, the CBC-MDV method relied on Ward-level livestock field officers (and in theory the district vet) who would need to travel to each village/sub-village to undertake the vaccinations. How was this done (what mode of transportation was used; a reference to walking and use of a motorbike was mentioned when describing the advertising campaign) and what were the costs associated with this compared to the pulse vaccination? I would assume that while these would typically be shorter trips, they would need to be undertaken more frequently, especially if on-demand vaccination is offered. While I understand that this was a compromise from the initial concept, the fact that law only permits vaccination by recognized staff is a common challenge in most rabies-endemic countries. This limitation is emphasized in lines 337 – 338 citing the lack of supervision from District veterinary officers as well as in lines 397-398 citing the long distances required to travel between villages for the advertising campaign. | Thank you for your observation and recommendation. CBC-MDV was delivered at the village-levels and relied on livestock field officers as vaccinators. The livestock field officers coordinated the organization of the campaigns and only travelled on the day of vaccination on their own motorbikes (all of them in the study had their own motor bikes and the project provided fuel) to vaccinate dogs in each village.  The means of transportation described applied to the OHCs and how they traveled during advertising. The OHCs also requested provision for transport in the face of the distance they had to cover and this is being considered in the next phase of the project.  The initial design of CBC-MDV anticipated that district-level livestock field officers would monitor the campaigns through phone calls and receiving of vaccination reports. The research also examined availability of infrastructure at the district offices, including means of transport for district officers to supervise the campaigns as part of their livestock field supervisions. This paper identified how these activities and arrangements can be improved (Table 6).  A full paper has been devoted to an economic analysis of the different strategies and is in preparation by the team. |
| 4)      Another question relating to the limitations of a pulse campaign versus CBC-MDV was the timing of the campaign – yet the CBC-MDV strategies were equally affected in terms of funerals, other commitments, poor weather, etc. If a Pulse campaign is well planned, these limitations can be easily avoided. | Thank you for your observation. This is only one aspect of how vaccination clinics can be impacted by local events. In terms of timing, the main difference is that while pulse is annual (with only one opportunity per village per year for vaccination to take place), CBC-MDV is continuous (with four campaigns in a year plus the option for on-demand). This means that, whilst both strategies can be affected by timing, the impact at the village level of disruption on a single day of a pulsed event would likely be more significant than the impact on a single day of CBC-MDV as this latter strategy provides more timepoint access than pulse vaccination. Lines |
| 5)      Line 310 – 313 mentions one ward from each district undertaking each of the three CBC-MDV strategies, while one ward from each district undertook the pulse campaign. However, in line 164 – 167, the authors mention that the pilot phase included 12 wards from each of the 3 districts (i.e., 36 wards in total). Please can you clarify the reasons as to why these figures differ. If the study was undertaken in 3 representative wards (one from each district) for each strategy, then this needs to be clarified in the methodology. | Thank you for pointing out the ambiguity. The following details “The pilot phase included 12 wards, four from each district (three delivered the CBC-MDV strategies and one delivered the pulse)”, have been added to clarify the statement in lines 175-177. |
| 6)      Line 401: For the advertising campaigns, 56% of OHC’s varied the advertising time to either morning or afternoon to attempt to reach more people. Was it the case that more people were reached at these times, or was this simply due to the inconvenience/other factors relating to deviating from the protocol? i.e., In those villages where the announcements were done at the "varied" time, were vaccination turnout rates lower? | Thank you for your observation. We do not actually know how many people heard or saw the advertising campaigns and although we could have used vaccination numbers as a proxy the sample size did not give us the power to do the comparisons inferentially. Instead, we focused on the reasons for varying the protocol, and in line with the aims of process evaluation, we sought to answer the question as to what works for whom and why, in order to optimize the design to fit well with daily routines of implementers and other contextual factors. This is explained in the discussion. Lines 692-698 |
| 7)      Table 5 shows the vaccination coverage for the pulse versus the 3 CBC-MDV strategies. While the pulse campaign achieved far lower coverage, was this as a result of poor planning and implementation rather than due to a reduced efficiency? The drop in vaccination coverage from the pulse campaign was only approximately 3% over the course of the year (assumedly due to births/new dogs). If the initial pulse campaign had reached the 70% (or more) coverage, then the overall vaccination coverage would not have decreased significantly, and the disease could be easily controlled. | Thank you for your observation.  The reviewer’s comment addresses two key issues around vaccination strategies, first the coverage achieved during campaigns and second, the decline in coverage between campaigns. We agree that if pulse vaccination is able to achieve 70% coverage, the decline in coverage through births (of susceptible) and deaths (of vaccinated dogs) should not result in coverage dropping below the critical threshold of 40%. However, the evidence shows that this level is often difficult to achieve due to the range of operational challenges (and it’s likely that these will apply widely across Africa) and that CBC-MDV offers an approach to achieving a coverage that can be more reliably sustained above 40%, through provision of continuous access to dog vaccination. |

Reviewer #2

| **Comments** | **Response** |
| --- | --- |
| 33. Suggest changing to “can eliminate rabies in dogs,”… | Thank you for your recommendation. The phrase “in dogs” is added, line 36 |
| 40. Define UK MRC | Thank you for your recommendation. The acronym “MRC” is defined, lines 43-44 |
| 48-49. Should this be in the methods rather than the results? | Thank you for your recommendation. However, in line with an objective of this paper to describe the development of CBC-MDV, we consider the decision “The final design included delivery by veterinary teams supported by village-level one health champions” as one of the outcomes of the development process of the intervention. |
| 69-85. The abstract discusses the 40% coverage level, but the author summary discusses the 70% coverage level. This confused this reviewer initially. | Thank you for your observation. The statement, “sustain vaccination coverage above 40%” is added to give context to the 70%, lines 77-78 |
| 165. A map or visual showing the structure of the wards, villages, and sub-villages could be useful if this would not breach ethics permits. It would be useful for readers not familiar with the region to understand the issues faced by the RC and OHC to travel between locations and gain support in geographically and potentially culturally separated groups. | Thank you for your recommendation.  We have included the map of the study area; showing districts, wards and villages (their surface areas). Subvillages are not shown because the current shape files for Tanzania do not have demarcations for subvillages.  The statement “The study wards were appreciably separated geographically and culturally” is added and its impact on campaigns is alluded to in line 177-178. |
| 180. Table 1 overlaps with Figure 1, and this reviewer did not find Figure 1 particularly useful, despite generally having a preference for figures over tables. This reviewer suggests to consider if Figure 1 is needed. | Thank you for your observation. While Fig 1 shows the phases of developing and testing the intervention prior to full scale evaluation using the MRC’s guidance, table 1 describes and documents key stakeholder engagement meetings and their outcomes |
| 207. Table 2. Remove comma from “24, fortnightly meetings” | Thank you for pointing out the error, the comma has been removed. |
| 317. Figure 2 also has significant overlap with a table, and there was no legend with the figure. | Thank you for your observation. Fig 2 shows the logic model for CBC-MDV, the legend is left in the body of the article on lines 332-333 per the PLOS guidelines, while table describes what the three strategies of CBC-MDV were and how they will be delivered generally |
| 321. Can Table 1 (supplementary file) be renamed to Table S1? | Thank you for pointing out the ambiguity. Table headings of supplementary files have been changed e.g. Table S1 |
| 345. The need to longer lead times should be a discussion point (maybe it already is?). | Thank you for your observation. The sentence “To enhance community involvement, project implementers must allow enough time for community entry and engagement processes to take effect before commencement of vaccination campaigns”, added to further highlight the importance of this, lines 639-642 |
| 359-364. This seems like it could be a major problem in repeated studies and in larger studies. Will working more directly with the mabalozis really solve the problem, or will some of them think that their social status entitles them to compensation without putting in an equivalent effort to the OHCs in this study? | Thank you for your observation. The aim of this process evaluation was to provide formative insights for optimizing the design before scale up and replication in other settings. Based on these findings, alternative community leadership (village chairpersons) are being tested in the full scale RCT (Table 6) |
| 412-414. How did the cracked pots affect vaccine effectiveness? Showing the temperature data from the pots would be helpful for understanding if this low-tech approach is feasible for large-scale vaccination campaigns. | The observation meant that water had to be added more frequently, or where they leaked badly, the RC had to store the vaccines in the pot of the nearest ward.  We refer to this briefly in the introduction, but have not discussed the implications fully here as the feasibility of storing vaccines in this low-tech passive cooling devices has been reported in detail by Lugelo et al., 2020. |
| 442-444. This seems strange than none of the OHCs gave out their mobile phone number and a large oversight not to have it included with information packets and advertising. A simple business card for the OHCs could have also partially solved this problem. | Thank you for your recommendation. We do agree with you. The OHCs did write the numbers on the posters they used to advertise the campaigns. But the posters disintegrated after a while so the team is considering printing the numbers of either RCs or OHCs on the vaccination cards that are issued to dog owners. Indeed, the placement of telephone numbers on the vaccination cards has been included as a recommendation in Table 6. |
| 442-480. The issue with aggressive dogs appears to be a major issue. It would be good to add a discussion section as to why oral rabies vaccines were not used, and/or whether they could be more effective in this situation. | Thank you for your recommendation. A paragraph has been added to discuss this point; “The challenge of dog aggression could be surmounted with use of oral vaccination approaches, either broadly or targeted at dogs that are difficult to restrain. Also, potentially, the CBC-MDV approach can be used to deliver oral rabies vaccination by communities adjacent to national parks to target wildlife (foxes, racoons, hyenas, wild dogs etc) that have frequent interactions with domestic dogs. However, studies conducted in Zimbabwe and Tunisia showed only 17% of 369 baits were chewed after 24 hours, indicating the need to distribute a large number of baits [34] and required more time to implement respectively [35]. Cost-effective evaluations of using oral bait versus mass injection must be made to inform the choice of approach”, lines 667-676 |
| 581-584 (Table 6). Table 6 is very challenging to interpret and has several open boxes. Please consider a complete redesign of this table to more effective convey the information. A take home message from Table 6 seems to be that people did not follow the instructions. A major question left at the end of this study is why should we expect a difference in subsequent trials? Several useful suggestions are offered, such as incorporating the vaccination dates into the local calendars, which could then lead to the vaccination dates becoming standardised over time. However, this information gets swamped in this complex table. | Thank you for your recommendation. Table 6 is now reorganized accordingly. |
| The comment that the OHCs “thought did more work than agreed upon” is concerning. Future studies will need to be very explicit in expected quantitative outputs to reduce the risk of alienating key partners and local vaccination champions. | Thank you for your recommendation. Yes, we agree with you. The CBC-MDV protocol prescribed OHCs assist only the vaccination campaigns of their villages but they responded to the work load at vaccination centers by collaborating with other OHCs of the ward. This is documented in the paper (lines 395-398). |
| 589. At what level is the “did not include direct involvement of community members” comment aimed? Does this refer to a person in each sub-village or more generally not working close enough with the community or mabolozis. | Thank you for your observation. The statement is reworded now with “representatives from the target villages” to give more clarity, lines 617-618 |
| 616-617. Are these references for dog vaccine studies? | Thank you for your question. The references are not specifically for dog vaccine studies but show there is scope for community participation in community-wide delivery of health interventions and the potential for dog vaccination campaigns and are very good examples of how community-based mass dog vaccination campaigns can be tailored |
| 638. Are RCs unpopular or not respected in the community due to collecting levies at auctions? I can’t think of many situations where people are happy to pay taxes/levies, so using an RC associated with levies might not be a good strategy. | Thank you for your observation. It is possible that attitudes towards RCs might be affected by the fact they collect levies. However, livestock owners also depend heavily on the services provided by the RCs and so RCs tend to be well respected and receive a lot of support. |
| 646-667. This is a very long paragraph that could be trimmed substantially. | Thank you for your recommendation. The paragraph is trimmed as much as possible, lines 692-705 |
| 689-694. Very long second sentence. Split to simplify. | Thank you for your recommendation. The sentence is now split, lines 713-717 |
| 708-724. This was a fantastic study with a great level of detail included in the manuscript, but was challenging to read at times. This conclusion needs to be much simpler so that anyone that reads part or all of the document retains and a simple take away story. | We are grateful for your recommendation. The writing has been edited and we hope/consider that the readability has been much improved.  The conclusion is trimmed |
| A major comment is that the readability of the tables and text needs to be improved. | The writing has been edited and we hope/consider that the readability has been much improved. |
| Several parts of the paper appear to be redundant, such as stating very similar information in the methods and results. | Thank you for your observation. As the paper describes outcomes of the development processes of the intervention, it has to report on the methodologies/ strategies of the intervention that were developed in the results section. That is, in the methods section, we described how the intervention was developed and, in the results section, we reported the intervention methodologies/ strategies that were developed. |
| The formatting of several tables makes it challenging to cross-reference information in separate columns; please do not let Tables break across pages, as this made Tables 2-3 difficult to interpret. | Thank you, we agree. Table 2 does not cross pages in the tracked changes version (lines 221-222). However, I could not find a way of fitting table 3 onto one page, even with a page break. I hope that when formatted for publication this will be avoided. |
| Additionally, the Supplementary File contains three tables, but they are not titled as supplementary tables (Table S1, Table S2, Table S3), which leads to confusion about the tables in the main manuscript. | Thank you for pointing out the error, the Table headings of supplementary files have been changed, e.g. Table S1 |
| The figures at the end of the main manuscript are in the order 1, 3, 4, 5, 2. Figure 2 has no legend and the legends for the other figure are inadequate. | Thank you for your observation. We revised the figure and their legends and re-ordered them per the PLOS guidelines. |
| A general comment throughout is that sentences should be shortened, and paragraphs should have at least three sentences | Thank you for your observation. The writing has been edited and we hope/consider that the readability has been much improved. |
| There are many non-standard acronyms in this manuscript; a short section at the beginning of the manuscript listing all the acronyms would be useful. | Thank you for your recommendation. A section for abbreviation is inserted with definitions of the following abbreviations:  CBC-MDV: Community-Based Continuous Mass Dog Vaccination  MDV: Mass Dog Vaccination  OHC: One Health Champion  RC: Rabies Coordinators, lines 70-74 |
| This strategy could be useful for many non-human diseases. It would be good to discuss how this approach could be used for non-rabies pathogens, and even for oral rabies vaccination campaigns for wildlife (fox, raccoon). | Thank you for your recommendation. A paragraph has been added to discuss this point; lines 667-676 |
| References Please review your reference list to ensure that it is complete and correct. If you have cited papers that have been retracted, please include the rationale for doing so in the manuscript text, or remove these references and replace them with relevant current references. Any changes to the reference list should be mentioned in the rebuttal letter that accompanies your revised manuscript. If you need to cite a retracted article, indicate the article's retracted status in the References list and also include a citation and full reference for the retraction notice. | Thank you for your recommendation. The relevance of all references has been checked and formatted in line with the style of PLoS NTD.  As far as we know, we have to cited any retracted article.  Thank you very much! |
